# SHP-1 Variants Broaden the Understanding of pH-Dependent Activities in Protein Tyrosine Phosphatases

**DOI:** 10.1021/jacsau.4c00078

**Published:** 2024-07-17

**Authors:** Ruidan Shen, Alfie-Louise R. Brownless, Nikolas Alansson, Marina Corbella, Shina C. L. Kamerlin, Alvan C. Hengge

**Affiliations:** † Department of Chemistry and Biochemistry, Utah State University, Logan, Utah 84322-0300, United States; ‡ School of Chemistry and Biochemistry, Georgia Institute of Technology, 901 Atlantic Drive NW, Atlanta, Georgia 30332-0400, United States; § Departament de Quımica Inorgànica i Orgànica (Secció de Quımica Orgànica) & Institut de Quımica Teòrica i Computacional (IQTCUB), Universitat de Barcelona, Martı i Franquès 1, 08028 Barcelona, Spain; ∥ Science for Life Laboratory, Department of Chemistry—BMC, Uppsala University, BMC, P.O. Box 576, S-751 23 Uppsala, Sweden

**Keywords:** protein tyrosine phosphatases, enzyme kinetics, protein dynamics, loop dynamics, pH dependency, molecular dynamics simulations

## Abstract

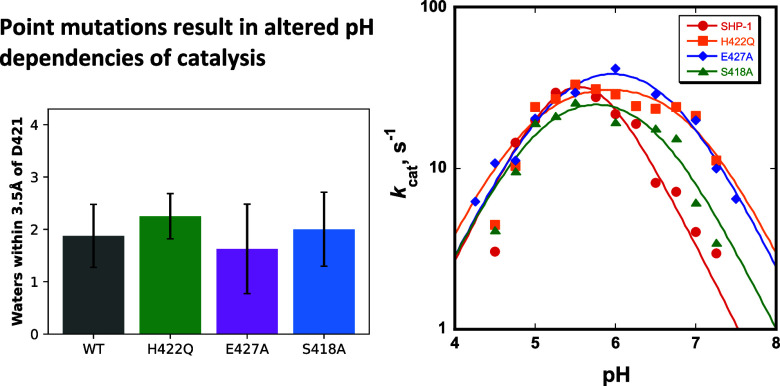

The protein tyrosine phosphatase (PTP) SHP-1 plays an important role in both immune regulation and oncogenesis. This enzyme is part of a broader family of PTPs that all play important regulatory roles in vivo. Common to these enzymes is a highly conserved aspartic acid (D421 in SHP-1) that acts as an acid/base catalyst during the PTP-catalyzed reaction. This residue is located on a mobile loop, the WPD-loop, the dynamic behavior of which is intimately connected to the catalytic activity. The SHP-1 WPD-loop variants H422Q, E427A, and S418A have been kinetically characterized and compared to those of the wild-type (WT) enzyme. These variants exhibit limiting magnitudes of *k*_cat_ ranging from 43 to 77% of the WT enzyme. However, their pH profiles are significantly broadened in the basic pH range. As a result, above pH 6, the E427A and S418A variants have turnover numbers notably higher than those of WT SHP-1. Molecular modeling results indicate that the shifted pH dependencies result primarily from changes in solvation and hydrogen-bonding networks that affect the p*K*_a_ of the D421 residue, explaining the changes in pH-rate profiles for *k*_cat_ on the basic side. In contrast, a previous study of a noncatalytic residue variant of the PTP YopH, which also exhibited changes in pH dependency, showed that the catalytic change arose from mutation-induced changes in conformational equilibria of the WPD-loop. This finding and the present study show the existence of distinct strategies for nature to tune the activity of PTPs in particular environments through controlling the pH dependency of catalysis.

## Introduction

Cellular pH varies from near neutrality in the nucleus and cytosol, compared to subcellular components such as the more basic mitochondria (pH ∼ 8)^1^ and the more acidic lysosome (pH ∼ 4.5).^2^ A recently developed method using nanowire probes found small differences in pH between the nucleus (6.92 ± 0.04) and the cytosol (7.11 ± 0.05).^3^ Differences in pH are also associated with several cellular processes.^2^ Examples include the intracellular acidification associated with apoptosis;^4,5^ reduced protein glycosylation that results from impaired acidification of the Golgi compartment;^6^ and pH-dependent regulation of synaptic activity in neurons. Intracellular pH varies from 0.3 to 0.5 units during the process of mitosis.^7^ Intracellular pH plays a large role in the activity of many proteins, including enzymes that exhibit pH dependencies on catalysis, providing clear connections with the associations between pH and cellular processes. Mutations can affect an enzyme’s pH dependency in several ways, and the potential effects of disease-associated mutations could arise from the resulting dysregulation of key enzymes in the cell.

The straightforward view is that the pH dependency of enzymatic catalysis arises from the p*K*_a_ values of catalytic residues taking part in the chemical steps. However, the pH dependency can vary between enzymes in the same family even when they share identical catalytic residues due to several factors that cause the kinetic p*K*_a_ values displayed in a pH-rate profile to be perturbed from their intrinsic thermodynamic values. For example, differences in the local environment can alter the p*K*_a_ values of catalytic residues. Additionally, the kinetic p*K*_a_ values reflected in a pH-rate profile are often distorted from the intrinsic, or thermodynamic, p*K*_a_ values of catalytic residues.^8−10^ For example, we recently showed that a point mutation to a noncatalytic residue in the protein tyrosine phosphatase (PTP) YopH does not significantly affect its maximal turnover number but broadens its pH-rate dependency, making the variant a much faster enzyme at low pH than the native enzyme.^11^ A computational analysis showed that this catalytic change arose from mutation-induced changes in conformational equilibria. Point mutations can also alter the p*K*_a_ of neighboring residues by altering the electrostatic environment or by inducing changes in hydrogen bonding and solvation networks. Because of such possibilities, the effect on enzymatic catalysis of any mutation is better assessed by assaying the rate across a pH range rather than at a single pH. Here, we report findings from a kinetic and computational investigation of point variants of another PTP, SHP-1, that were reported to exhibit faster catalysis than the wild-type (WT) at pH 5.^12^

**Figure 1:**
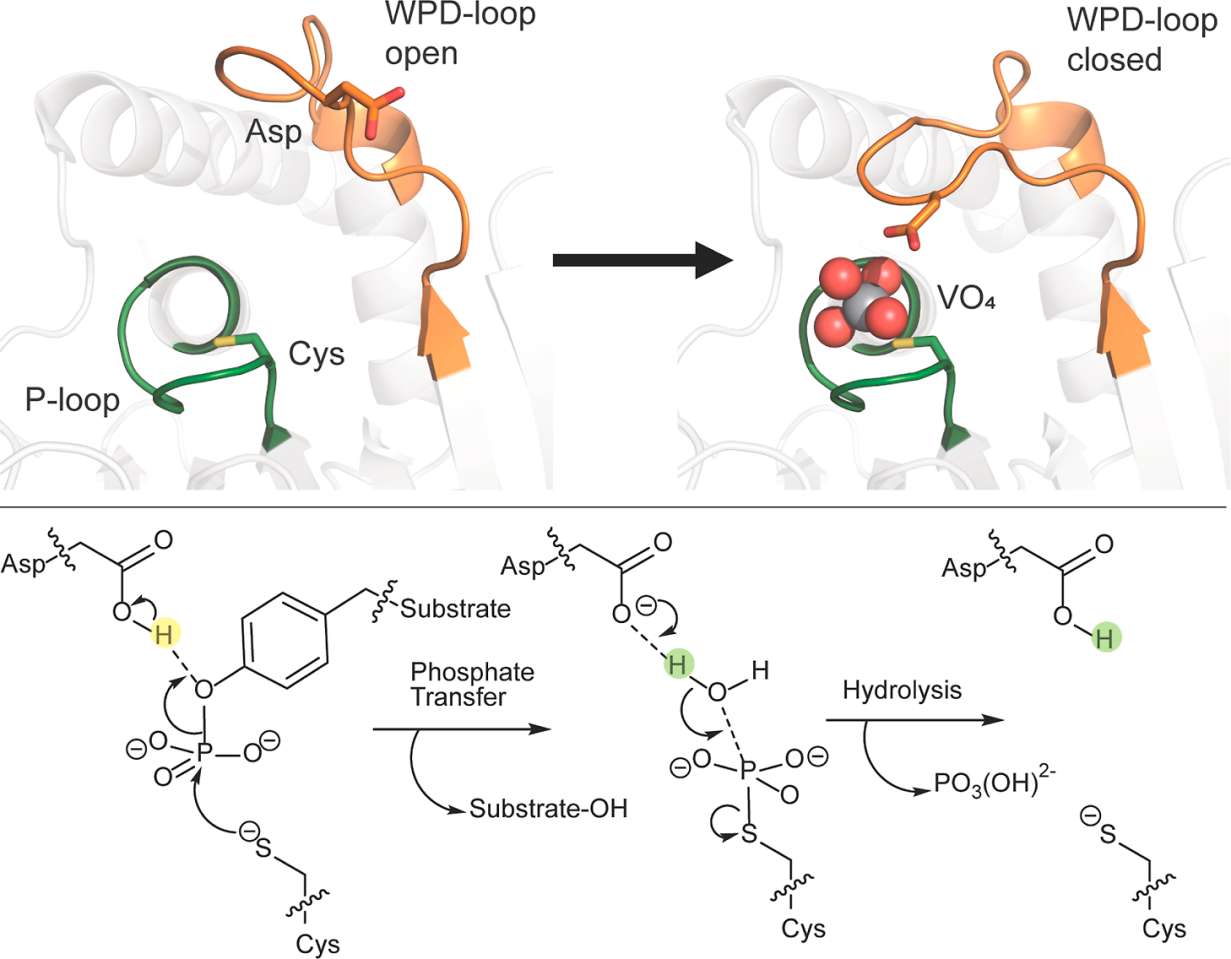
PTPs utilize a two-step mechanism involving the P-loop (shown in green) and the WPD-loop (shown in orange). The WPD-loop exhibits two distinct conformations: an open, noncatalytic conformation and a closed, catalytically active one, shown here complexed with the transition state analogue vanadate. The WPD-loop closes toward the P-loop, which brings the conserved aspartic acid into position to protonate the leaving group, followed by a subsequent step where the same residue acts as a general base to activate a water molecule in the hydrolysis of the phospho-cysteine intermediate. The vanadate ion is shown in spheres, and key catalytic side chains are shown in sticks. The WPD-loop is shown in orange, and the P-loop is shown in green. Reproduced with permission from ref (16) Copyright 2022, Royal Society of Chemistry. Originally published under a CC-BY-NC 3.0 license.

The catalytic activities of PTPs have bell-shaped pH dependencies and show optimal activity within a narrow range of pH.^13,14^ The bell-shaped pH-rate profiles result from a mechanism that requires specific protonation states of two conserved catalytic residues, an aspartic acid and a cysteine^15^ (Figure 1). The first step of the PTP mechanism requires a protonated aspartic acid and a deprotonated cysteine nucleophile. Conformational changes in the PTP active site also contribute to activity and the formation of the catalytically competent enzyme–substrate complex. Classical PTPs exhibit two major conformers that differ in the major orientation of a solvent accessible loop motif, the WPD-loop. In substrate-bound classical PTPs, a WPD-loop closed, catalytically active conformation predominates, while in the free enzymes, the WPD-loop is primarily found in an open, catalytically nonproductive conformation. Catalysis in PTPs has been correlated to protein motions. In particular, the dynamics of the WPD-loop involving the chemical steps and the variability of these dynamics with pH can affect the populations of catalytically active enzyme–substrate complexes. Mutations that affect loop equilibrium can thus alter the pH-rate profile of catalysis in PTPs.^11^

SHP-1 plays a critical role in human immune systems, especially in regulating B-cell and T-cell signal transduction pathways.^17,18^ Mutations and impaired SHP-1 activities have been linked to the murine motheaten disease and familial hemophagocytic lymphohistiocytosis,^19,20^ featuring SHP-1 as an intriguing research target for clinical studies. The phosphatase activity of the full-length SHP-1 is autoinhibited at rest by the insertion of an SH2 domain to its active site, while the enzyme resumes the catalytically active conformation upon cellular stimulation.^21−24^ Similar to two of the most characterized PTPs, YopH and PTP1B, SHP-1 contains a mobile WPD-loop and a static P-loop. The overall secondary structure of SHP-1 is highly superimposable to that of PTP1B according to X-ray studies, especially the signature active site motifs, the WPD-loop, and the P-loop.

X-ray data indicate that SHP-1 shares a mobile WPD-loop with those of other classical PTPs. It has been inferred that SHP-1 and other PTPs with mobile WPD-loops exhibit the same correlation between loop dynamics and catalysis as seen in PTP1B and YopH.^25,26^ This study investigated the pH-dependent enzymatic activities of several point variants of the catalytic subunit of SHP-1 that were reported to exhibit elevated turnover rates and efficiencies^12,27^ at pH 5 in solutions containing 40% glycerol. Glycerol raises both *k*_cat_ and *K*_M_ of SHP-1 catalysis and increases susceptibility to proteolysis, effects tentatively ascribed to relaxation of structural components.^28^ We were interested in whether these variant SHP-1 proteins had higher activities in general and how their pH dependencies of catalysis compared to the native enzyme. Our kinetic studies were carried out in aqueous solution, more reflective of biological conditions and to avoid complicating effects of glycerol. Glycerol is a potent viscosigen, and the cosolvent will also affect pH by altering the p*K*_a_ of buffering species and affect the p*K*_a_ and nucleophilicity of catalytic residues. These kinetic studies are supported by molecular dynamics (MD) simulations of WT and variant SHP-1 in the unliganded and phosphoenzyme intermediate states to explore the impact of the substitutions on the dynamics of the WPD-loop. Curiously, our computational results show minimal impact of the mutations on loop dynamics, suggesting that the observed shifts in pH dependency are due to chemical effects (primarily through modulating the p*K*_a_ of the catalytic aspartic acid), in contrast to YopH where altered pH dependencies were shown to be due to altered dynamics of the WPD-loop.^11^

## Materials and Methods

### Chemicals

Dithiothreitol (DTT), kanamycin monosulfate, lysozyme, and ampicillin (AMP) were purchased from GoldBio. Protease inhibitors aprotinin, pepstatin A, and leupeptin were purchased from Sigma-Aldrich. HisPur Ni-NTA Resin was purchased from Thermo Fisher. All other buffers and reagents were purchased from Sigma-Aldrich or Thermo Fisher. The substrate *p*-nitrophenyl phosphate (*p*NPP) was synthesized using a published method.^29^

### Expression and Purification

The plasmid pEt-32 encoding WT SHP-1 was provided by Alicea-Velazquez, and the plasmid pEt-21 (+) encoding variants SHP-1 H422Q, SHP-1 E427A, and SHP-1 S418A were purchased from Twist Bioscience.

The DNA was transformed into BL21-DE3 cells and grown overnight at 37 °C on an LB culture plate containing 100 ng/μL of kanamycin monosulfate for WT SHP-1 or 100 ng/μL of ampicillin for the SHP-1 variants. One colony was selected and placed into 10 mL of SOC media containing 100 ng/μL of kanamycin monosulfate or ampicillin and grown overnight. The following morning, 1 L of LB media containing 100 ng/μL of kanamycin monosulfate or ampicillin was inoculated with the 10 mL of overnight growth and shaken at 170 rpm at 37 °C until the OD_600nm_ reached 0.6–0.8. After the optimal OD was reached, the 1 L growth was induced by 0.1 mM isopropyl β-d-thiogalactoside (IPTG) and shaken at 170 rpm and room temperature overnight. The cells were harvested by centrifugation at 12,000*g* for 30 min at 4 °C and stored at −80 °C.

WT SHP-1 cells were thawed on ice and resuspended in 10 mL of equilibration buffer, consisting of 25 mM Tris pH 8.0, 1 mM DTT, and 200 mM NaCl with 2 mg of lysozyme, 0.5 mg/mL of aprotinin, 0.7 mg/mL of pepstatin A, and 0.5 mg/mL of leupeptin. SHP-1 variant cells were thawed and resuspended in 50 mM bis-Tris at pH 6.5, 1 mM EDTA, 3 mM DTT, and 10% glycerol with the same protease inhibitors. The cells were lysed by sonication at 70% amplitude for 30 s and then mixed on ice for 1 min and repeated 5–6 times until completely lysed. The cell lysate was centrifuged at 4 °C at 30,000*g* for 30 min.

The WT SHP-1 lysate was purified via a 1 mL HisPur Ni-NTA column. Cleared lysate was applied to the column resin, followed by a wash step with 10 column volumes of equilibration buffer and 10 column volumes of wash buffer, consisting of 25 mM Tris pH 8.0, 1 mM DTT, 200 mM NaCl, and 20 mM imidazole. Elution for WT SHP-1 was processed using 15 mL elution buffer containing 25 mM Tris pH 8.0, 1 mM DTT, 200 mM NaCl, and 500 mM imidazole. Eluted fractions were tested with *p*NPP for phosphatase activity. Fractions that showed activity were pooled and dialyzed in 2 L equilibration buffer with 0.5 mg TEV protease overnight at 4 °C with gentle stirring. Dialyzed and untagged SHP-1 was applied to a second Ni-NTA column, followed by 10 column volumes of equilibration buffer and 10 column volumes of wash buffer. The flow-through containing untagged SHP-1 was pooled and concentrated to <12 mL, loaded onto a pre-equilibrated HiLoad 26/60 Superdex 200 prep grade column (GE), and purified with 25 mM Tris pH 8.0, 150 mM NaCl, and 0.5 mM TCEP. Fractions were assayed with *p*NPP for activity and purity on a 15% SDS-PAGE gel. Pure protein was concentrated to 10–20 mg/mL, diluted with 10% glycerol, frozen with liquid nitrogen, and stored at −80 °C in aliquots.

All SHP-1 variants, SHP-1 H422Q, SHP-1 E427A, and SHP-1 S418A were purified via a 5 mL HiTrap Q HP column attached above a 5 mL HiTrap SP HP column by using an FPLC filtration system. Both columns were equilibrated with lysis buffer. The cell lysate was loaded onto the columns, the HiTrap Q HP column was removed after loading, and the HiTrap SP HP column was washed with lysis buffer until the absorbance at 280 nm was baselined. Elution for SHP-1 variants was processed using a 100% gradient with elution buffer containing 500 mM NaCl, 50 mM bis-Tris pH 6.5, 1 mM EDTA, 3 mM DTT, and 10% glycerol. Eluted fractions exhibiting absorbance at 280 nm were collected and tested with *p*NPP for phosphatase activity. Fractions that showed activity were assayed for purity on a 15% SDS-PAGE gel.

The active fractions were pooled and concentrated to <12 mL, loaded onto a pre-equilibrated HiLoad 26/60 Superdex 200 prep grade column (GE), and purified using 10 mM bis-Tris buffer pH 6.5, with 25 mM NaCl, 0.2 mM EDTA, and 3 mM DTT. Fractions were assayed with *p*NPP for activity and purity on a 15% SDS-PAGE gel. Pure protein was concentrated to 10–20 mg/mL and either kept on ice to immediately set up crystal trays or diluted with 10% glycerol and frozen with liquid nitrogen and stored at −80 °C in aliquots.

### Steady-State Kinetics

Steady-state kinetic parameters were measured at 25 °C. Concentrated protein aliquots were thawed on ice and diluted with a buffer base mix (BBM) containing 50 mM sodium acetate, 100 mM Tris, and 100 mM bis-Tris from pH 4.0 to pH 7.5. This buffer system maintains a constant ionic strength throughout the pH range examined. A 50 mM solution of *p*NPP was prepared in the BBM. The reactions were run on a 96-well plate using substrate concentrations from 0.76 to 10.61 mM. Reactions were allowed to proceed for 2–8 min for WT SHP-1 and the SHP-1 variants. The reactions were quenched using 50 μL of 2.5 M NaOH, and the amount of the product *p*-nitrophenol was assayed from the absorption at 400 nm using the molar extinction coefficient of 18,300 M^–1^ cm^–1^. Reaction blanks were made using identical conditions replacing the enzyme with buffer to correct for nonenzymatic hydrolysis of the substrate. The amount of product released and elapsed time were used to calculate the initial rates. These data were fitted to the Michaelis–Menten equation to obtain steady-state kinetic parameters. Kinetic data were obtained on both variants as a function of pH to obtain pH-rate profiles which were fitted to eqs 1 and 2. In eq 2, *K*_S2_ was set to the second ionization constant of the substrate *p*NPP (p*K*_a_ = 4.96). These equations relate the dependence of the observed values of *k*_cat_, or *k*_cat_/*K*_M_, to their maximal, or limiting, values as a function of pH, where catalysis is dependent on two ionizable enzymatic residues, one protonated and the other deprotonated.
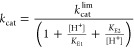
1
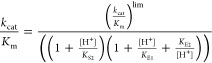
2

### Molecular Dynamics Simulations and Simulation Analysis

MD simulations were performed using the GROMACS simulation package v. 2022.05,^30^ together with the ff14SB^31^ force field and TIP3P^32^ water model for the parameters of protein atoms and water molecules, respectively. We used two initial crystal structures (PDB ID: 4HJP(33) and 4GRZ(33)) of the catalytic domain of the SHP-1 enzyme, which correspond to WPD-loop open and closed conformations of the enzyme, respectively. GROMACS-compatible topology files using the ff14SB force field^31^ to describe the protein were generated by tLEaP and ParmEd v. 4.1.0.^34^ Simulations were performed of both the WT enzyme and the H422Q, E427A, and S418A variants, in both the unliganded form of the enzyme and at the phosphoenzyme intermediate preceding the rate-limiting hydrolysis step of the reaction^26^ (Figure 1), as in prior work.^16^ This corresponded to 16 simulation systems in total (4 variants, WPD-loop open/closed and liganded/unliganded forms from each state).

Parameters for the phosphoenzyme intermediate were obtained from prior work, see the Supporting Information of ref (26). Point mutations were introduced into the WT structures using PyMol.^35^ Residues 315–317 in PDB ID: 4HJP(33) were missing from the crystal structure and were manually introduced by overlaying with PDB ID: 4GRZ.^33^ Missing residues at the N- and C-termini were omitted from our simulations (Gly241, Ser242, Val525, and Gln526 in both structures, and Gln527 and Ser528 in PDB ID: 4GRZ(33)). PROPKA 3.0^36,37^ was used to check for anomalous p*K*_a_ values for ionizable residues, and, based on this evaluation, all systems were kept in their standard protonation states at physiological pH. The protein was then placed inside a truncated octahedral water box of TIP3P^32^ water molecules, extending 10 Å from the solute in all directions, and the system was neutralized with Na^+^ counterions.

Energies were minimized by using the steepest descent algorithm. The system was heated to 300 K over 100 ps using velocity rescaling.^38^ This was followed by 100 ps *NPT* production at 300 K and 1 atm, using a Parinello–Rahman barostat.^39^ For each system, 8 replicas of 1.5 μs long MD simulations were carried out, for a total of 12 μs for each of the 16 systems and 192 μs total simulation time over all systems. The convergence of the simulations can be seen in Figure S1. As parts of SHP-1 (including, in particular, the N-terminal region of the protein) are highly flexible, we obtain individual trajectories with high root-mean-square displacement (RMSD) for several systems; however, the average RMSD is overall stable for all systems on the performed simulation times.

MD simulations were run with a time step of 2 fs (by making use of the SHAKE algorithm for constraining hydrogen bonds^40^) and a cutoff of 10 Å for nonbonded interactions, while long-range electrostatics were calculated with the particle-mesh Ewald method.^41^ Temperature and pressure were controlled by means of the modified Berendsen thermostat (with velocity rescaling^38^ of 0.1 ps) and a Parinello–Rahman barostat^39^ (with pressure fluctuations at equilibrium every 2 ps). All simulation analyses were carried out using CPPTRAJ^42^ and MDAnalysis^43,44^ based on snapshots saved every 250 ps of MD simulation time.

### Empirical Valence Bond Simulations

Empirical valence bond (EVB) simulations^45^ of WT SHP-1 and variants were performed at the phosphoenzyme intermediate preceding the rate-limiting hydrolysis step, using the same protocol as in prior work,^16,26,46^ using the OPLS-AA force field^47^ and the TIP3P water model.^32^ PDB ID 4GRZ(48) was used to describe the closed catalytic state, while the different variants were generated in silico by truncation of the corresponding side chains or substitution for the highest probability rotamer using the Dunbrack 2010 Rotamer Library.^49^ A list of ionized residues in our simulations necessary for describing the system using the Surface Constrained All Atom Solvent Model, SCAAS,^16,26,46,50^ (see prior work^16,26,46^) is found in Table S1. All simulations were performed in 30 individual replicates per system, with each trajectory involving an initial 30 ns of equilibration followed by 10.2 ns of EVB simulation (51 EVB mapping frames × 200 ps simulation time per frame), as in prior work,^16,46,51^ to a total simulation time of 1.206 μs per system and 4.824 μs cumulatively across all systems. Convergence of the EVB equilibration runs is shown in Figure S2. The EVB mapping parameters shown to obtain calculated activation free energies are identical to those used in prior work.^16,46,51^

## Results and Discussion

### Kinetics

A previous kinetic study of a set of SHP-1 alanine variants reported higher phosphatase activities than the WT enzyme, speculated to arise from increased WPD-loop flexibility and stability.^12^ One of these mutations was to the residue immediately following the general acid D421, and it was speculated that the side-chain length of the D+1 residue affected the catalytic rate by altering the dynamic rate of the WPD-loop.^12,27^ There is precedent for a mutation in the D+1 position affecting turnover by altering the WPD-loop dynamics. In the D+1 position in PTP1B, the mutation F182Q results in a decrease of about an order of magnitude in *k*_cat_ with no shift in the pH optimum but a broader maximum than the native enzyme and retention of the basic limb.^11^ Because of the effect on the pH dependency of catalysis in that case, we sought to investigate some of the previously reported SHP-1 variants across a pH range to more broadly examine the effect of the mutations. We collected and compared kinetic data for the catalytic activities of three SHP-1 variants, S418A, H422Q, and E427A, with those of WT-SHP-1. Residues S418 and E427 reside on the N-terminal and C-terminal hinges of the WPD-loop, respectively, and H422 is the D+1 residue.

**Figure 2:**
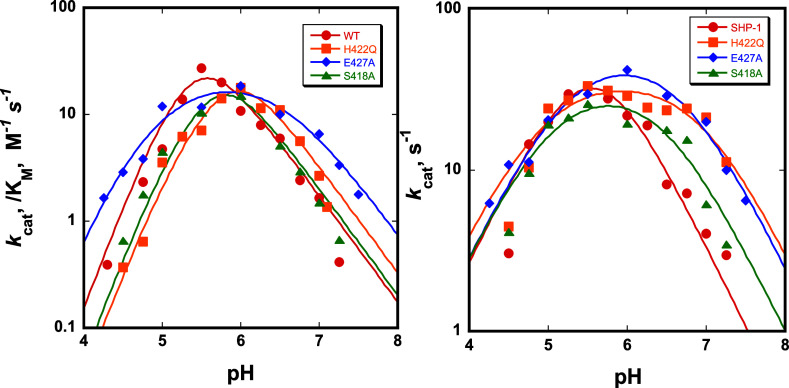
pH dependencies of *k*_cat_/*K*_M_ (left) and of *k*_cat_ (right) of WT SHP-1 and the H422Q, E427A, and S418A variants in complex with the substrate *p*NPP. Each of the variants exhibits higher turnover numbers at basic pH than WT.

Figure 2 shows the pH-dependent catalytic activity with the substrate *p*NPP for WT SHP-1 and the variants H422Q, E427A, and S418A. The parameter *k*_cat_/*K*_M_ reflects the part of the overall mechanism up to and including the first irreversible step, formation of the phosphoenzyme intermediate, which is the first step in Figure 1. The overall rate-determining step in the SHP-1 reaction, reflected in *k*_cat_, is the hydrolysis of this intermediate, in which D421 acts as a base to deprotonate a nucleophilic water molecule.^28,52^ The pH dependency of *k*_cat_/*K*_M_ is broader in the variants, which is reminiscent of the variability of pH dependencies within the PTP family. For example, YopH catalysis shows a narrower pH profile compared to PTP1B. These PTPs share catalytic residues but differ in residues neighboring the catalytic ones and within their WPD-loops. In the present case, the broader pH profile reflects the fact that the E427A variant has higher catalytic efficiency at low and high pH. The acidic limbs of the *k*_cat_ pH-rate profiles and the maximal activities showed modest changes between the variants and the native enzyme, but the basic limbs show more significant effects. Each of the variants has turnover numbers above pH 6 higher than that of the native enzyme. At pH 7, H422Q and E427A have turnover numbers approximately 5-fold higher than WT SHP-1.

Focusing on *k*_cat_, which reflects the rate-determining step in turnover, the variants show alterations in the pH optimum, in their pH-independent limiting *k*_cat_ values, and the kinetic p*K*_a_ values obtained from fits to eq 1 (Table 1). The kinetic p*K*_a_ values for the aspartic acid in the variants are each ∼1 unit higher than that for the WT, reflected in their broader pH-rate profiles. The native SHP-1 has a kinetic p*K*_a_ of the aspartic acid of 5.6, while in the variants, it is increased to 6.9, 6.7, and 6.5 for H422Q, E427A, and S418A, respectively. Though the variants and WT have similar activities at their optimal pH, all of the variants have faster turnover at pH > 6. In contrast, the pH-independent limiting *k*_cat_ values show a minimal difference between the different enzyme variants, an observation also supported by EVB simulations (Table 1). For comparison, Table 1 lists the previously reported *k*_cat_ values obtained in acetate buffer at pH 5.0 with 40% added glycerol. The p*K*_a_ of carboxylic acids increases in nonaqueous solvents and in cosolvent mixtures. The p*K*_a_ of acetic acid in glycerol/water mixtures has not been reported to our knowledge but is increased by a full unit in 50% ethanol.^53^ It is possible that the aqueous glycerol conditions were at an effective pH of 6 or above, in the range where our aqueous solution *k*_cat_ values for the variants are higher than WT (Figure 2, right).

**Table 1:** Kinetic p*K*_a_ Values, pH-Independent Limiting *k*_cat_ Values, and pH Optima for WT SHP-1 and Variants Obtained from Fits of *k*_cat_ Data to Eq 1^a^

	WT SHP-1	SHP-1 H422Q	SHP-1 E427A	SHP-1 S418A
*k*_cat_ (s^–1^) (40% glycerol, pH 5)	57.6 ± 1.5,^b^ 125.8^c^	295.5^c^	120.7 ± 0.6^b^	145.8 ± 11.4^b^
*k*_cat_^lim^ (s^–1^)	73 ± 16	35 ± 4	56 ± 7	32 ± 6
Δ*G*^‡^_calc_ (kcal mol^–1^)^d^	15.4 ± 0.2	15.6 ± 0.3	15.3 ± 0.2	15.5 ± 0.2
p*K*_a1_	5.2 ± 0.2	4.9 ± 0.2	5.2 ± 0.1	5.1 ± 0.2
p*K*_a2_	5.6 ± 0.2	6.9 ± 0.2	6.7 ± 0.1	6.5 ± 0.2
pH optimum	5.36	5.94	5.96	5.77

a The p*K*_a1_ value arises from the cysteine nucleophile and p*K*_a2_ from the aspartic acid. Shown here for comparison are reported *k*_cat_ values (s^–1^) in 40% glycerol solution, pH 5.^12,27^

b Data from ref (12).

c Data from ref (27).

d Calculated activation free energies, in kcal mol^–1^, presented as average values and standard error of the mean across 30 independent EVB trajectories propagated for each system, performed as described in the Materials and Methods.

### Structural Based Analysis

**Figure 3:**
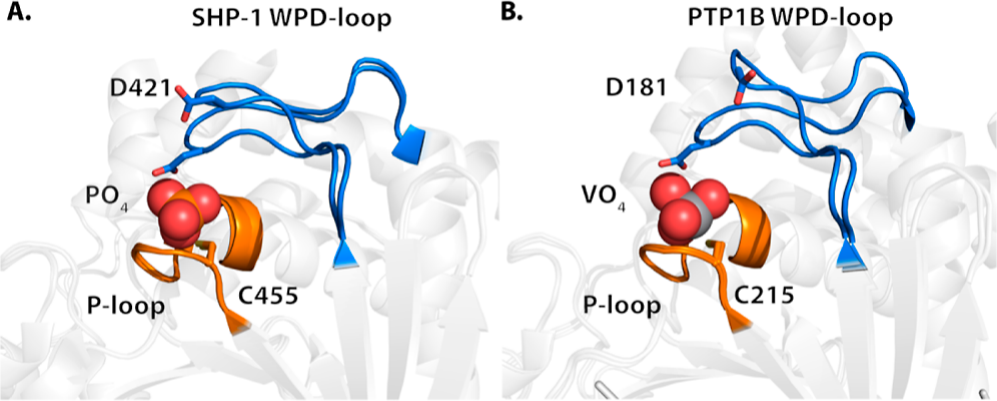
WPD-loop of SHP-1 exhibits a smaller conformational change compared to that of PTP1B. The catalytic residues cysteine and aspartic acid are shown in sticks; the WPD-loops are in blue, and the P-loops are in orange. (A) WT SHP-1 ligand-free and phosphate-bound structures with loop open (upper) and closed (lower) states PDB IDs: 4HJP(33) and 4HJQ,^33^ respectively; (B) WT PTP1B ligand-free and vanadate-bound structures with loop open (upper) and closed (lower) states PDB IDs: 2CM2(54) and 3I80,^55^ respectively.

Point mutations on noncatalytic WPD-loop residues in PTPs rarely affect the secondary structures of the active sites; however, differences in the WPD-loop dynamics are likely to be observed.^11,16^ A previous study of point variants of corresponding residues on the WPD-loops of PTP1B and YopH demonstrated that a single mutation changes the pH dependence of catalysis, loop dynamics, and loop equilibrium. Residues S418, H422, and E427 are located on the N-terminal, central portion, and C-terminal of the WPD-loop in SHP-1. Although SHP-1 has a modest difference in the backbone positioning of its WPD-loop compared to YopH and PTP1B (Figure 3), residues S418, H422, and E427 are likely to experience, as well as affect, significant conformational dynamics between loop closed and open forms. In PTP1B, the central portion of the WPD-loop and residues E186 and S187 exhibits relatively high mobility compared to the rest of the loop.^26^ Due to the high sequence identity and structural similarity of the WPD-loop of SHP-1 and PTP1B, it would not be unreasonable to assume that SHP-1 exhibits similar dynamics and flexibility of the loop to PTP1B, and that these SHP-1 mutations might alter pH dependency by changing loop conformational equilibria.^11^

**Figure 4:**
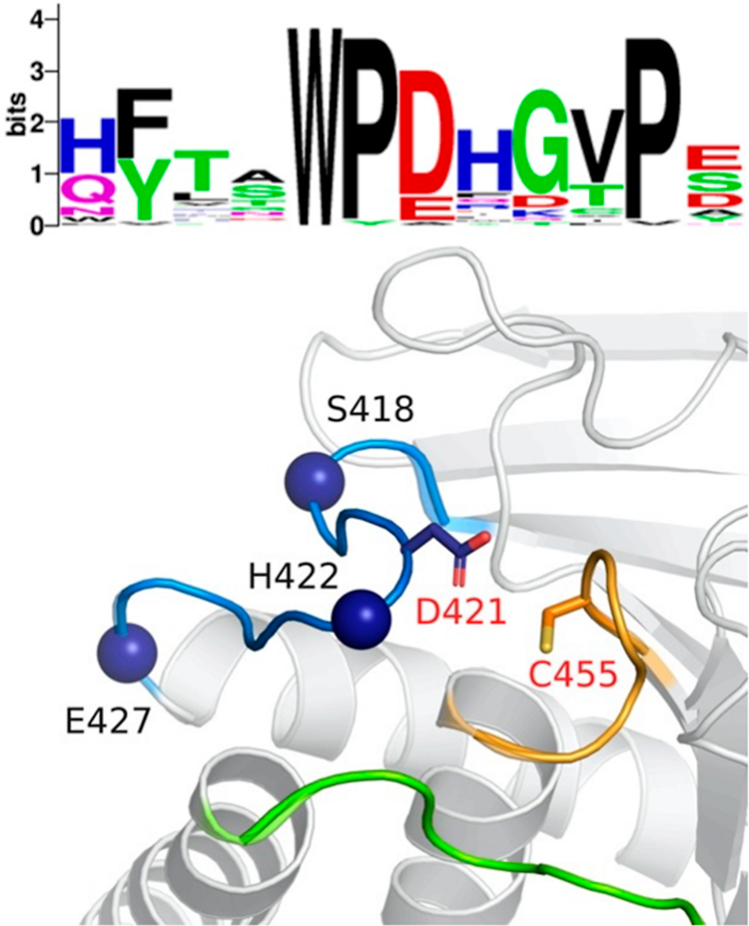
Top: sequence logo of the WPD-loop based on a library of classical active PTPs, as defined by Chen et al.,^56^ as well as the canonical YopH sequence. The multiple sequence alignment (MSA) was generated using the MUSCLE Web server utility,^57^ and gappy positions (>50%) were stripped. The resulting MSA was converted into a sequence logo using Weblogo,^58^ only visualizing the 12 WPD loop residues. Reproduced from ref (16) with permission from the Royal Society of Chemistry. Originally published under a CC-BY-NC-CD license. Bottom: location of the residues mutated in this study is shown by spheres in the WPD-loop (blue). The P-loop with the nucleophilic C455 is colored orange, and the Q-loop is shown in green.

**Figure 5:**
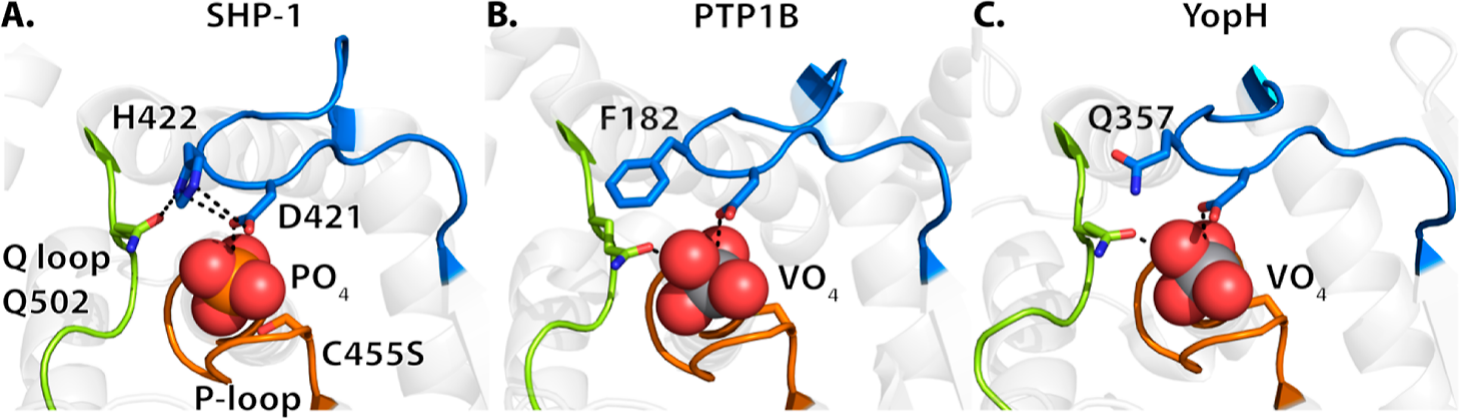
Stabilization interactions provided by H422 in SHP-1 allow for direct coordination between the WPD-loop (blue) and the Q-loop (green) upon substrate binding, further stabilizing the WPD-loop closed conformation. The respective D+1 residue in PTP1B (F182) and YopH (Q357) are shown in sticks (SHP-1 PDB ID: 4HJP,^33^ PTP1B PDB ID: 3I80,^55^ and YopH PDB ID: 2I42(59)).

SHP-1 and most members of the classical PTP family have conserved histidines in the D+1 position (Figure 4). In the loop open form, the imidazole group and the backbone amide of H422 form hydrogen bonds with the carboxylate group of D421; while in the loop closed form, compared to the D+1 residue in PTP1B (F182) and YopH (Q357), the side chain of H422 provides extra hydrogen bonds to stabilize the side-chain conformation of D421 and the conserved glutamine (Q502) in SHP-1 (Figure 5). The interactions provided by H422 coordinate the WPD-loop and the Q-loop, which further stabilize the closed-conformation of the WPD-loop upon ligand binding.

### Molecular Dynamics Simulations

**Figure 6:**
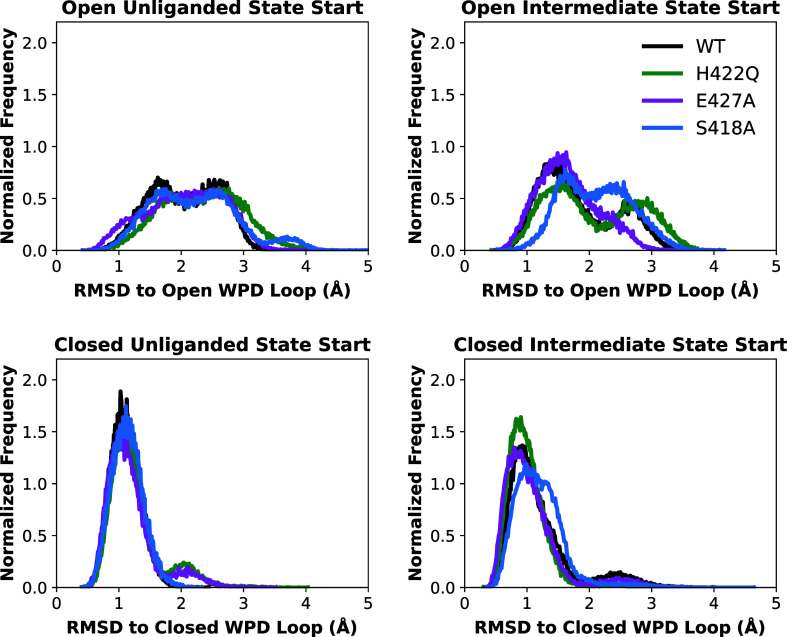
Histograms of the root-mean-square deviations (rmsd, Å) of the C_α_-atoms of the SHP1 WPD-loop relative to the starting (crystal structure) position used for each set of simulations of the WPD-loop open unliganded, WPD-loop closed unliganded, WPD-loop open phosphoenzyme intermediate, and WPD-loop closed phosphoenzyme intermediate states of SHP1 and variants (see Materials and Methods for crystal structure used). Data was obtained from 8 × 1.5 μs independent MD simulations of each system.

Our prior structural and computational work indicated that shifts in pH dependency upon mutation of YopH were due to a shift in the conformational equilibrium of the WPD-loop toward a loop-closed conformation.^11^ To examine whether the altered pH-rate profiles of the SHP-1 variants in this study result from alterations in WPD-loop conformational equilibria or other causes, simulations of the WT enzyme as well as the H422Q, E427A, and S418A variants were performed in both unliganded and phosphoenzyme intermediate forms of each variant and with simulations initiated from both the loop open and loop closed conformations of these variants. For each system, we evaluated the rmsd of both the C_α_-atoms of just the WPD-loop of the different SHP-1 variants (Figure 6) as well as the root-mean-square fluctuations of these atoms across the full enzyme scaffold (Figure S3). Based on this data, there are no statistically significant differences between WT SHP-1 and the variants as determined by pairwise *t*-tests conducted at each residue index followed by a Benjamin–Hochberg correction^60^ (apart from small local differences in flexibility in residue A513 E427 and P428 between the WT and E427A variant). This is in contrast to our prior studies of PTP1B and YopH, where a single point swap between T177G (PTP1B) and G352T (YopH) on the WPD-loop significantly affected loop dynamics and the pH dependency of catalysis.^11^ For comparison, the PTP1B F182Q point mutation similarly has no significant impact on loop dynamics, but in this case, the pH dependency of catalysis is unaffected.^16^

We have successfully used the EVB approach^45^ to model chemistry in both WT PTP1B and YopH and variants.^16,26,46^ In the present case, while the H422Q, E427A, and S418A mutations all impact the rates of catalysis by the SHP-1 catalytic domain,^12,27^ from a thermodynamic perspective, the difference in activation free energy between the variants is very small (within 0.5 kcal mol^–1^ change compared to WT), and thus EVB (or other) simulations of the chemical step of catalysis are unlikely to capture the origins of these small energy differences. Nevertheless, our EVB simulations (Table 1) do indicate that within this resolution, the differences in calculated activation free energies between the variants are minimal, in agreement with the pH-independent limiting *k*_cat_ values (Table 1).

**Figure 7:**
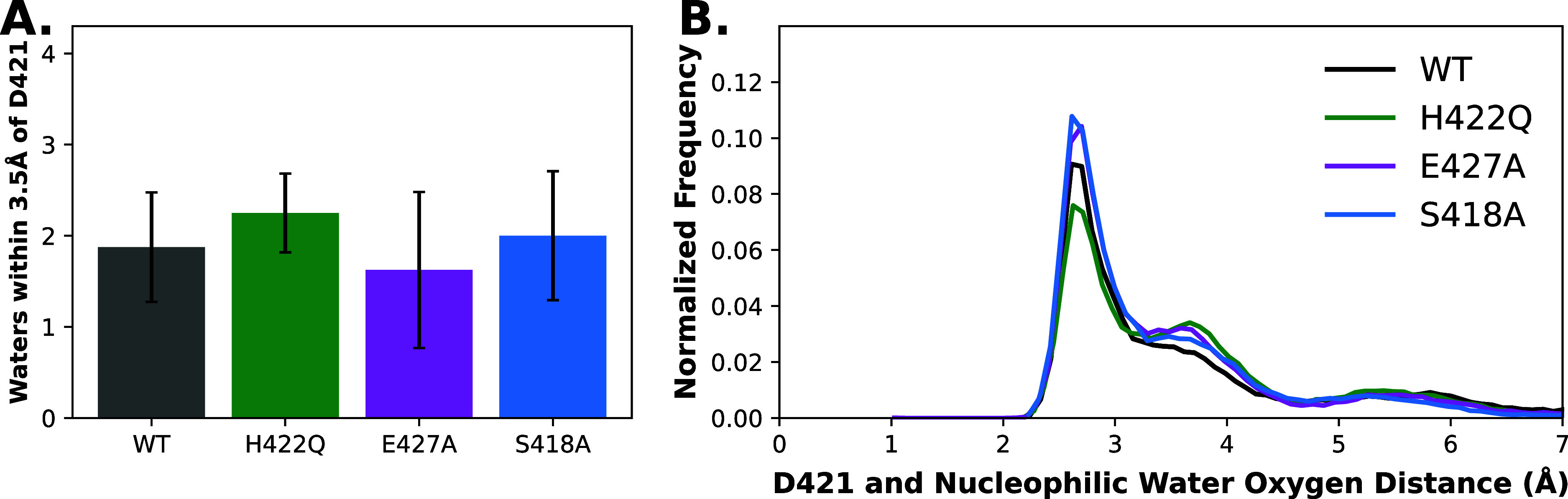
(A) The number of water molecules within 3.5 Å of the side chain of the catalytic acid D421 based on distances computed between the Cγ-atom of D421 and the oxygen atom of each water molecule. Errors bars represent the standard deviation of the calculation. (B) Distribution of the distance between the closest side-chain oxygen of the general acid, D421, and the oxygen of the nucleophilic water molecule (selected based on closest P–O_wat_ distance and closest to linear in-line O_wat_–P–O_lg_ angle to the phospho-cysteine group, i.e., the water molecule best aligned for nucleophilic attack in any given simulation frame). Data presented is based on analysis of 8 × 1.5 μs independent MD simulations of WT SHP1 and variants, at the WPD-loop closed phosphoenzyme intermediate state of each enzyme. The raw data for this figure are shown in Table S2.

Interestingly, examination of the number of water molecules within 3.5 Å of the side chain of D421 (measured as the distance between the C_γ_-atom of the D421 side chain and the oxygen atoms of the surrounding water molecules) during our simulations of the phosphoenzyme intermediate with a closed WPD-loop (Figure 7A and Table S2) suggests subtle differences in solvation of the aspartic acid that could rationalize the differences in p*K*_a_ of this residue.

The data shown in Figure 7A is further bolstered by examination of solvent density in the active site of each SHP-1 variant during our simulations (Figure S4), which we calculated using the Density Analysis tool of the MDAnalysis package.^43^ Water densities were calculated over the whole trajectory of our simulations of the closed phosphoenzyme intermediate of each enzyme, focusing on the catalytic aspartic acid, and the volumes in Figure S4 represent water density hotspots. As can be seen from this analysis, there exist (as would be expected) areas of high water density near each oxygen atom associated with the phospho-cysteine residue, with subtle differences in water density between the different SHP-1 variants. While these qualitative changes are more challenging to quantitatively link directly to changes in enzyme activity, it appears that the point mutations reduce the number of water density hotspots behind the D421 side chain, which would in turn contribute to elevating the p*K*_a_ of this side chain, as suggested by the kinetic p*K*_a_ measurements shown in Table 1.

Furthermore, the mean P–O_nuc_ distance during our simulations of WT SHP1 and variants in the phosphoenzyme intermediate state is very subtly shifted to a tighter P–O distance, which would be expected to be favorable for catalysis, although this difference is within the standard deviation of the calculations (Figure 7B and Table S2).

**Figure 8:**
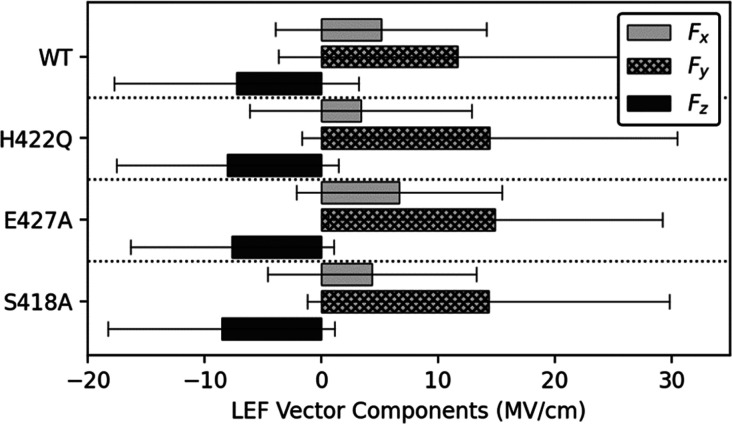
Averaged LEF vector components collected across the closed phosphoenzyme intermediate MD simulation data. Notably, the *F*_*z*_ force vectors point up the S–P bond within the phospho-cysteine residue and do not show substantial differences between the WT and variant forms of SHP-1. Error bars represent the ± standard deviation. Due to the magnitude in inherent variation within these electric field calculations, the results do not indicate notable differences between the variant and WT structures.

As it is possible that these subtle structural shifts could also impact the electric field acting on the phosphate group and generated from the protein and solvent environment, we also performed quantum chemical calculations of the local electric field (LEF) force vectors applied onto the phosphoenzyme intermediate in WT and SHP1, variants following a published protocol.^61^ This analysis was based on PDB snapshots extracted every 200 frames of our corresponding MD simulations of each system at the WPD-loop closed phosphoenzyme intermediate (which corresponds to the rate-limiting step of catalysis), resulting in approximately 240 snapshots per structure. To facilitate direct comparison between systems, these snapshots were then aligned to a common axis, in which the *F*_*z*_ force vectors were forced to point in parallel to the P–S bond of the phospho-cysteine residue. Subsequently, the phospho-cysteine residue was removed from the analysis, and NPA charges (from natural population analysis) were collected using the Atomic Charge Calculator II Web server^62^ at the HF/6-31G(d)/PCM level of theory. The electric field component of vectors applied onto the phosphate atom was then calculated at each PDB snapshot using Coulomb’s law, with the resulting data shown in Figure 8.

Our electric field calculations suggest general increases in the *F*_*y*_ components in the variant forms of SHP-1 compared to WT, along with some variation in the *F*_*x*_ components. The *F*_*z*_ components do not appear to be affected by mutations. However, due to the inherent variability attributed to these calculations, there is no evidence that the mutations dramatically change any one component of the LEF vector. Rather, these electric field calculations showcase mild shifts in magnitudes of vector components between starting states, which could contribute to the differences in catalytic rate due to subtle differences in conformation of active site residues.

**Figure 9:**
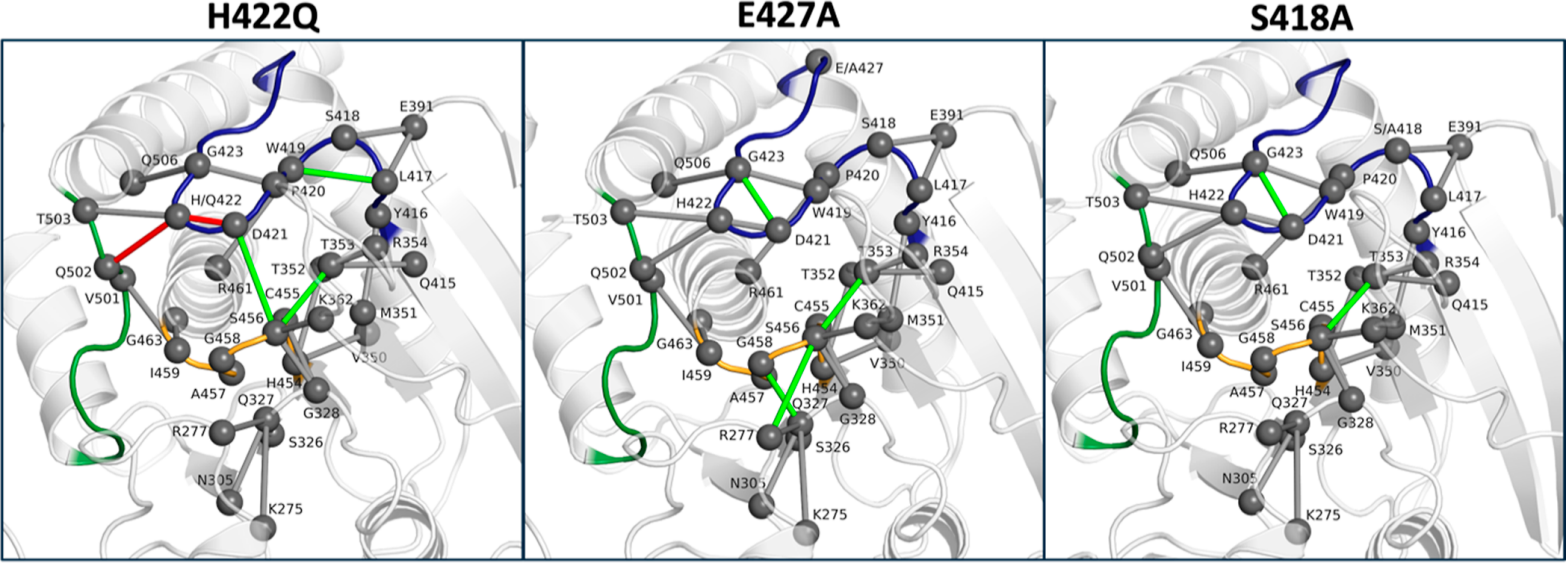
Hydrogen-bonding networks during simulations of WT and SHP1 variants. Analysis was performed using KIN on simulations of the catalytically relevant closed phosphoenzyme intermediate state. Gray connections indicate hydrogen bonds found within at least 25% of simulation time of the WT enzyme, which are conserved in simulations of SHP1 variants, which are then projected onto structures of each of the individual variants. Green connections indicate additional hydrogen bonds found within at least 25% of the simulation time for the corresponding variant that were not observed in the WT enzyme. Red connections indicate the disappearance of WT hydrogen bonding due to the introduction of the mutation in each system. The WPD-, P-, and Q-loops are shown here in blue, orange, and green, respectively.

As a final point of analysis, we considered whether the substitutions introduced on the SHP-1 WPD-loop are disrupting key hydrogen bonding interactions in the enzyme. To explore this, we conducted H-bond network analysis of our simulations of the WPD-loop closed phosphoenzyme intermediate state of WT SHP-1 and variants, using the contact calculation functionality of Key Interaction Networks (KIN).^63^ Specifically, we analyzed all hydrogen bonds within the immediate active site, in addition to all WPD-loop residues, taking into account interactions with occupancy times of at least 25% of the simulation. We focused on interactions that are (1) conserved in simulations of WT and variant SHP-1 (Figure 9, gray), (2) emerge in only simulations of the variants and are absent in WT SHP-1 (Figure 9, green), and (3) are present in WT SHP-1 but are lost in simulations of the variants (Figure 9, red).

From this analysis, it can be seen that in most cases, the introduction of WPD-loop point mutations stabilizes additional active-site hydrogen bonds compared to the WT enzyme. The H422Q variant, in particular, exhibits a loss of the H422–D421 and H422–Q502 interactions, consistent with experimental observations and as would be expected from mutating this residue. This mutation further allows for new W419–L417 and D421–S456 interactions to surface to compensate for this. R277–S456 interactions are present during E427A simulations. Both the E427A and S418A variants allow for the D421–G423 interaction to the surface. Interestingly and universally, all variants allow for the formation of new S456–T353 interactions, which are not seen with a large prominence within WT simulations. These adjustments in hydrogen bonding networks likely allow for modified positioning of the D421 side chain with respect to neighboring residues, facilitating the adjustments in p*K*_a_ and thus differences in catalytic rate at varying pH that is observed experimentally. Furthermore, increased hydrogen bonding interactions exhibited by E427A and S418A variants, especially, likely reduce active-site flexibility, allowing for more effective catalysis despite variations in pH.

## Conclusions

A prior investigation of these SHP-1 variants at pH 5.0 in a medium containing 40% glycerol observed faster rates than the WT enzyme, which were hypothesized to arise from enhanced WPD-loop dynamics.^12,27^ This study shows that the variants do not actually have broadly faster rates in aqueous solution, but rather, their pH dependencies have been altered, and, as a result, some of the variants exhibit faster turnover than WT at pH > 6. This is reflected in higher experimentally measured kinetic p*K*_a_s of catalytic residue D421 (Table 1).

Furthermore, our MD simulations indicate that there are no statistically significant differences in WPD-loop dynamics between these enzyme variants. Rather, our simulations of the different SHP-1 suggest subtle differences in solvation of the aspartic acid side chain (Figures 7 and S4 and Table S2), coupled with similarly subtle differences in the electric field on the phosphate ion at the phosphoenzyme intermediate (Figure 8), and, finally, altered hydrogen bonding networks in the variants as a result of the loop mutations (Figure 9).

Thus, the modeling results and structural analysis suggest that the origins of shifted pH dependencies among these SHP-1 variants result primarily from changes in solvation and hydrogen-bonding networks that affect the p*K*_a_ of the D421 residue rather than changes in loop dynamics, as was previously suggested.^12,27^ This observation in turn explains why the changes in the pH-rate profiles for *k*_cat_ in these variants are observed on the basic limb. These findings contrast with the observations from mutations in YopH and PTP1B, whose pH dependencies for *k*_cat_ were more broadly affected on both acidic and basic limbs, and where differences in the pH dependency of catalysis could be directly linked to altered dynamics of the WPD-loops of these enzymes.^11^

In our prior study,^11^ rmsd analysis of WPD-loop motion in WT and T177G/G352 PTP1B/YopH (respectively) showed that the T ↔ G substitution contributed to both stabilizing the WPD-loop closed conformational state and destabilizing the WPD-loop open conformational state. In the case of the G352T YopH variant, the loss of conformational flexibility upon mutating glycine to threonine was shown to rigidify the N-terminal portion of the WPD-loop (where residue 352 is located) as well as nearby structural elements, stabilizing the closed conformation of the loop. In the open conformation of the loop, this substitution conversely increased the flexibility of the central and C-terminal portions of the loop, as well as the adjacent (and connecting) α4-helix, destabilizing the loop-open conformation. The corresponding mutation in PTP1B, T177G, was shown to have a more complex effect because substituting threonine to a more conformationally flexible glycine side chain destabilized positions 177 and 178 in both loop-open and loop-closed conformations, but this was compensated by rigidification of the central and C-terminal portions of the loop for both loop conformations. This is basically the mirror image of what we observe in YopH for the reverse mutation; in the case of PTP1B, this effect is more pronounced in the closed conformation, leading to a more stable closed conformation and a more flexible open conformation; thus, it overall has the same result as in YopH, but for opposite reasons.

In the case of SHP-1 in the current study, we considered 3 mutations: H422Q, E427A, and S418A. These are either adjacent to or closer to the center of the WPD-loop (Figure 4) than the T/G substitution we previously studied in PTP1B and YopH.^11^ As shown in Figure 9, H-bonding interactions that are lost upon substitution are compensated for by the emergence of new H-bonding interactions that, in particular, in the E427A and S418A variants appear to stabilize the loop, likely leading to the retention of WT-like dynamics, in contrast to our prior work. Furthermore, not only does the H422Q substitution create new H-bonding networks (Figure 9), the S418A and E427A substitutions create additional space in the active site pocket, which in turn alters the solvation patterns of the active site (Figures 7 and S4). Clearly, the fact that such subtle differences in loop sequence lead to such major differences in loop dynamics poses a challenge for engineering such dynamics in a targeted fashion.

The results here combined with our previous work show the presence of multiple avenues by which noncatalytic residues can affect the pH dependency of catalysis, not only in PTPs, but also in enzymes in general. Aside from the three highly conserved residues that give the WPD-loop its name, PTPs have significant sequence variation within this loop. These differences can produce changes in pH dependency from alteration in loop dynamics, as our past work revealed, or, as shown in this work, via changes to the solvation and hydrogen bonding networks of catalytic residues. These avenues for tuning activity provide a potential explanation for the observation that, while all PTPs exhibit bell-shaped pH dependencies, their optima range from ∼4.5 to ∼7.5. Both phenomena provide both a means for nature to tune the activity of enzymes in particular environments and a tool for evolutionary development and differentiation. The findings apply beyond the PTP family to enzymes in general by demonstrating avenues through which point mutations can alter an enzyme’s activity in pH-dependent cellular processes, potentially with pathogenic consequences. The findings also emphasize the need to characterize the catalytic effect of any enzyme mutation beyond the common practice of making comparative kinetic measurements at a single pH.

## Supporting Information Available

Root-mean-square deviations of backbone C_α_-atoms from simulations; simulated water densities and protonation states of residues; and number of water molecules within 3.5 Å of D421 (PDF)
