# Supplementary material for: SHP-1 Variants Broaden the Understanding of pH-Dependent Activities in Protein Tyrosine Phosphatases
Source: JACS Au. 2024 Jul 17;4(8):2874–85. doi: 10.1021/jacsau.4c00078 (PMC11350601; doi:10.1021/jacsau.4c00078)
Supplement: Supplementary file 1 — au4c00078_si_001.pdf [file au4c00078_si_001.pdf]

Supporting Information for:

SHP-1 Variants Broaden the Understanding of pH-Dependent Activities in Protein Tyrosine Phosphatases

Ruidan Shen,[a,‡] Alfie-Louise R. Brownless,[b,‡] Nikolas Alansson,[b] Marina Corbella,[c,d] Shina C. L. Kamerlin*[,b,d], and Alvan C. Hengge*[,a]

[a] Department of Chemistry and Biochemistry, Utah State University, Logan, Utah 84322-0300, USA.

[b] School of Chemistry and Biochemistry, Georgia Institute of Technology, 901 Atlantic Drive NW, Atlanta, Georgia 30332-0400, USA.

[c] Departament de Química Inorgànica i Orgànica (Secció de Química Orgànica) & Institut de Química Teòrica i Computacional (IQTCUB), Universitat de Barcelona, Martí i Franquès 1, 08028 Barcelona, Spain.

[d] Science for Life Laboratory, Department of Chemistry − BMC, Uppsala University, BMC, Box 576, S-751 23 Uppsala, Sweden.

[‡] Both authors contributed equally to this work.

Corresponding author email addresses:

skamerlin3@gatech.edu

alvan.hengge@usu.edu

Table of Contents

Supplementary Figures……………………………………………………………………S3

Figure S1: Root mean square deviations (RMSD, Å) of backbone C_α-atoms across SHP1 MD simulations for wild-type and mutants.

Figure S2: Root mean square deviations (RMSD, Å) of backbone C_α-atoms across SHP1 EVB equilibration simulations for wild-type and mutants.

Figure S3: C_α-atom root mean square fluctuations (RMSF, Å) between loop-open and closed conformation of wild-type SHP-1 and mutants.

Figure S4. Visualization of calculated water-density hotspots in our simulations.

Supplementary Table 1…………………………………………………………………S7

Table S1: Protonation states of ionizable residues during SHP1 EVB simulations.

Table S2: Number of water molecules within 3.5Å of the side-chain of the catalytic acid D421, and distance between D421 and the nucleophilic water, for wild-type SHP1 and mutants.

Supplementary References……………………………………………………………S9

Supplementary Figures

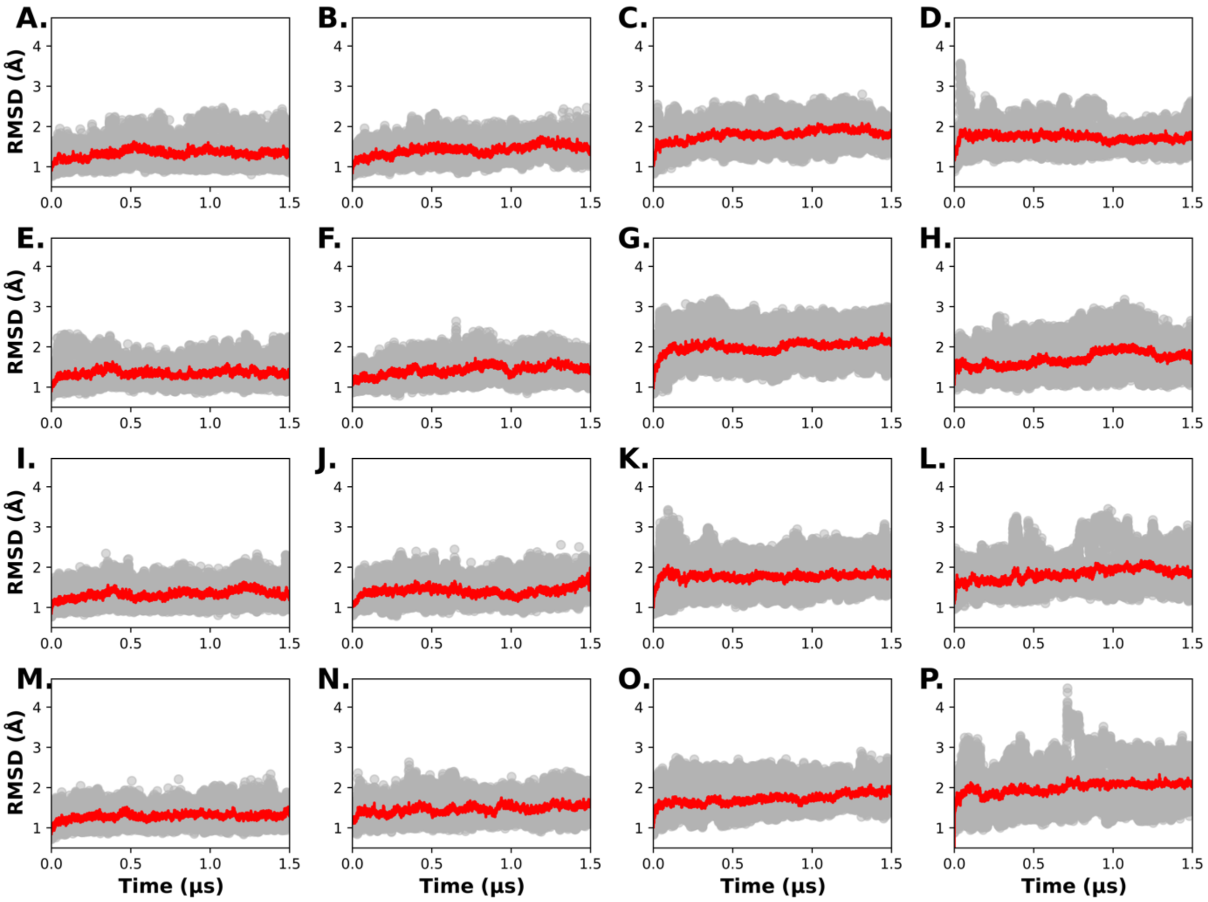

Figure S1. Root mean square deviations (RMSD, Å) of backbone C_α-atoms across SHP1 MD simulations (8 x 1.5μs for each system) of (**A-D**) wild-type SHP-1, as well as the (**E-H**) H422Q, (**I-L**) E427A, (**M-P**) and S418A SHP-1 variants, during simulations initiated from either a (**A**, **E**, **I**, **M**) closed unliganded starting state, (**B**, **F**, **J**, **N**) closed phosphoenzyme intermediate starting state, (**C**, **G**, **K**, **O**) open unliganded starting state, or a (**D**, **H**, **L**, **P**) open phosphoenzyme intermediate starting state. The RMSD is measured in reference to the WPD-loop conformation (open or closed) from which the simulation was initialized (based on PDB IDs: 4GRZ [1] for the closed, and 4HJP [2] for the open conformations, respectively). Red lines denote the average across all replicas, while gray dots describe results from each individual run. For all calculations, residues

1-17 were omitted due to the high flexibility of the N-terminus within the open conformation. Further, one trajectory of the unliganded E427A variant was discarded from further analysis and replaced with an additional trajectory due to system instability, the origins of which could not be obviously identified during the simulations.

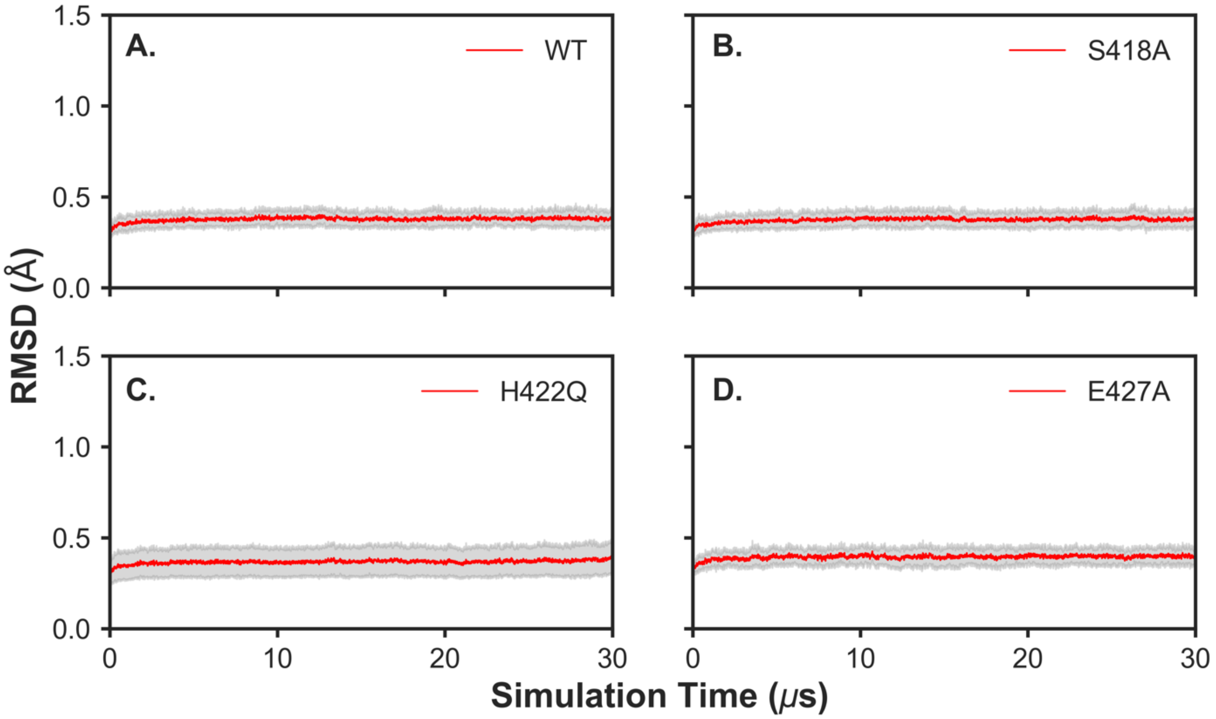

Figure S2. Root mean square deviations (RMSD, Å) of all backbone heavy atoms from EVB equilibration simulations of the hydrolysis step of *p*NPP, as catalyzed by (**A**) wild-type SHP-1, and the (**B**) S418A, (**C**) H422Q and (**D**) E427A SHP-1 variants. Data was collected every 10 ps from 30 replicas of 30 ns length each. The solid lines show rolling averages of the RMSD over all 10 replicas, and the shaded regions show the corresponding standard deviations in these values.

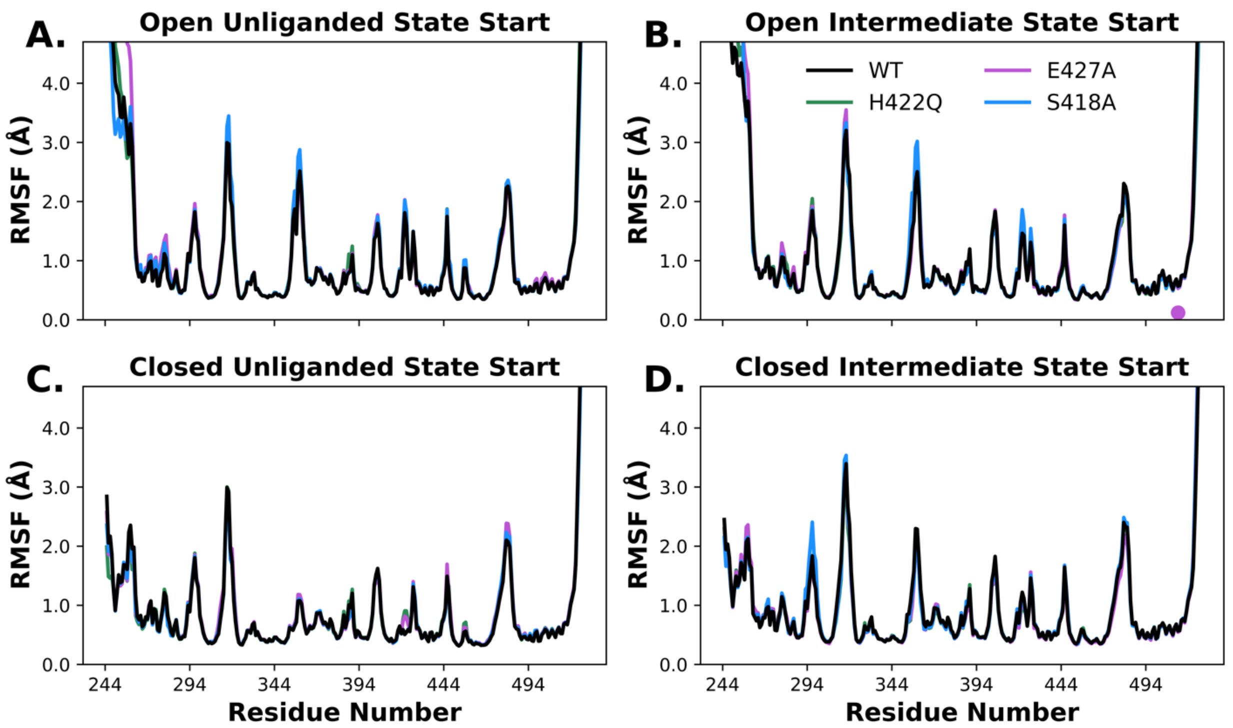

Figure S3. Comparisons between calculated per residue C_{α}-atom root mean square fluctuations (RMSF, Å) of the full catalytic domains of wild-type SHP-1 as well as the H422Q, E427A and S418A variants, during simulations initiated from the (**A**) open unliganded, **(B)** open intermediate, (**C**) closed unliganded and **(D)** closed phosphoenzyme intermediate states of each variant. The red dots below the curve indicate RMSF values that have been identified as statistically significant, based on a two-sample t-test and a Benjamini-Hochberg correction [3] using a false discovery rate of 0.05 ($p < 0.05$). Only the E427A mutant exhibits RMSF values that are statistically different form the wild-type at position A513.

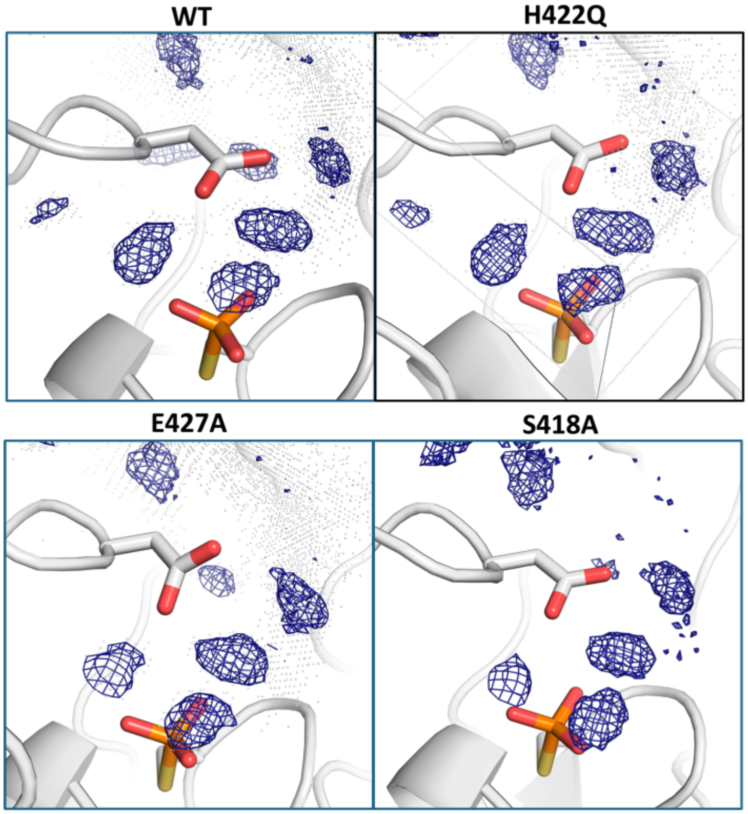

Figure S4. Visualizations of calculated water density hotspots present throughout conventional MD simulations of the WPD-closed phosphoenzyme intermediate in WT SHP1 and variants. Areas of high water density are present adjacent to the phospho-cysteine residue due to the strong negative charge associated with the phosphate group. Additional water clusters are present surrounding the D421 acid and generally do not appear to differ among mutants. Mutants appear to show a slight reduction in hotspot presence around the D421 acid, which could contribute to observed differences in kinetic pK_a (**Table 1**) However, the E427A and S418A mutants appear to show a reduction of water density near the left side of the WPD loop.

Supplementary Tables

Table S1. List of ionized residues and histidine protonation patterns used in our EVB simulations of SHP1 wild-type and variants S418A, H422Q and E427A.[a]

Residue Type	Residue Number
Asp	285, 336, 421, 436, 469
Glu	251, 257, 331, 344, 355, 357, 386, 391, 427, 504
Arg	264, 277, 294, 354, 360, 395, 461, 494, 497
Lys	273, 275, 279, 321, 358, 362, 393, 508
His-ε	262, 286, 387, 447, 454
His-δ	413, 422

[a] The ionizable residues that fell outside of the explicit simulation sphere were kept in their neutral forms to avoid system instabilities created by having changed residues outside the water droplet (this is standard practice for such simulations). All other residues were kept in their usual ionization state at physiological pH. In the case of the histidine side-chains, His-ε and His-δ indicate histidine side-chains protonated at the $N_{\varepsilon 2}$ and $N_{\delta 1}$ nitrogen atoms, respectively.

Table S2. The number of water molecules within 3.5Å of the side-chain of the catalytic acid D421 (water count) and the corresponding D421 – nucleophilic water molecule distance (D421 water distance) from simulations of wild-type SHP1 and variants.[a]

Enzyme Variant	Water Count	D421 Water Distance (Å)
Wild-Type	1.9 ± 0.6	3.97 ± 1.79
H422Q	2.2 ± 0.4	3.65 ± 1.17
E427A	1.6 ± 0.9	3.67 ± 1.47
S418A	2.0 ± 0.7	3.55 ± 1.33

[a] The water count was calculated based on distances between the C_{γ}-atom of D421 and the oxygen atom of each water molecule in its proximity. The D421 water distance was based on selecting the distance between the closest side-chain oxygen atom of D421 and the oxygen of the nucleophilic water molecule (selected based on closest $P\text{-}O_{wat}$ distance and closest to linear in-line $O_{wat}\text{-}P\text{-}O_{lg}$ angle to the phosphocysteine group, *i.e.* the water molecule best aligned for nucleophilic attack in any given simulation frame). Data presented as averages and standard deviations based on analysis of 8 x 1.5 μs independent MD simulations of wild-type SHP1 and variants, at the WPD-loop closed phosphoenzyme intermediate state of each enzyme.

References

1. Alicea-Velázquez, N. L.; Jakoncic, J.; Boggon, T. J., Structure-Guided Studies of the SHP-1/JAK1 Interaction Provide New Insights into Phosphatase Catalytic Domain Substrate Recognition. *J. Struct. Biol.* **2013,** *181*, 243-251.

2. Alicea-Velazquez, N. L.; Boggon, T. J., Shp Family Protein Tyrosine Phosphatases Adopt Canonical Active-Site Conformations in the Apo and Phosphate-Bound States. *Protein Pept. Lett.* **2013,** *20*, 1039-1048.

3. Benjamini, Y.; Hochberg, Y., Controlling the False Discovery Rate: A Practical and Powerful Approach to Multiple Testing. *J. R. Stat. Soc. B* **1995,** *57*, 289-300.
